# *catena*-Poly[[aqua­[3-(3-pyrid­yl)acrylato]gadolinium(III)]-bis­[μ-3-(3-pyrid­yl)acrylato]]

**DOI:** 10.1107/S1600536808012981

**Published:** 2008-05-10

**Authors:** Zhi-Hui Qiu, Fu-Pei Liang, Qing-Feng Ruan, Zi-Lu Chen

**Affiliations:** a College of Chemistry and Chemical Engineering, Guangxi Normal University, Guilin 541004, People’s Republic of China; b Faculty of Earth Sciences, China University of Geosciences, Wuhan 430074, People’s Republic of China; c Department of Resources and Environmental Engineering, Guilin University of Technology, Guilin 541004, People’s Republic of China

## Abstract

In the title compound, [Gd(C_8_H_6_NO_2_)_3_(H_2_O)]_*n*_, the gadolinium(III) ion is coordinated by eight carboxyl­ate O atoms and one water mol­ecule. The carboxyl­ate ligands bridge pairs of gadolinium(III) ions, forming a zigzag chain along [100]. Hydrogen bonds link the chains into sheets parallel to (001).

## Related literature

For related literature, see: Ayyappan *et al.* (2001 ▶); Gunning & Cahill (2005 ▶); Zhang *et al.* (2000 ▶). For related structures, see: Liu *et al.* (2004 ▶, 2006 ▶); Zhou *et al.* (2004 ▶).
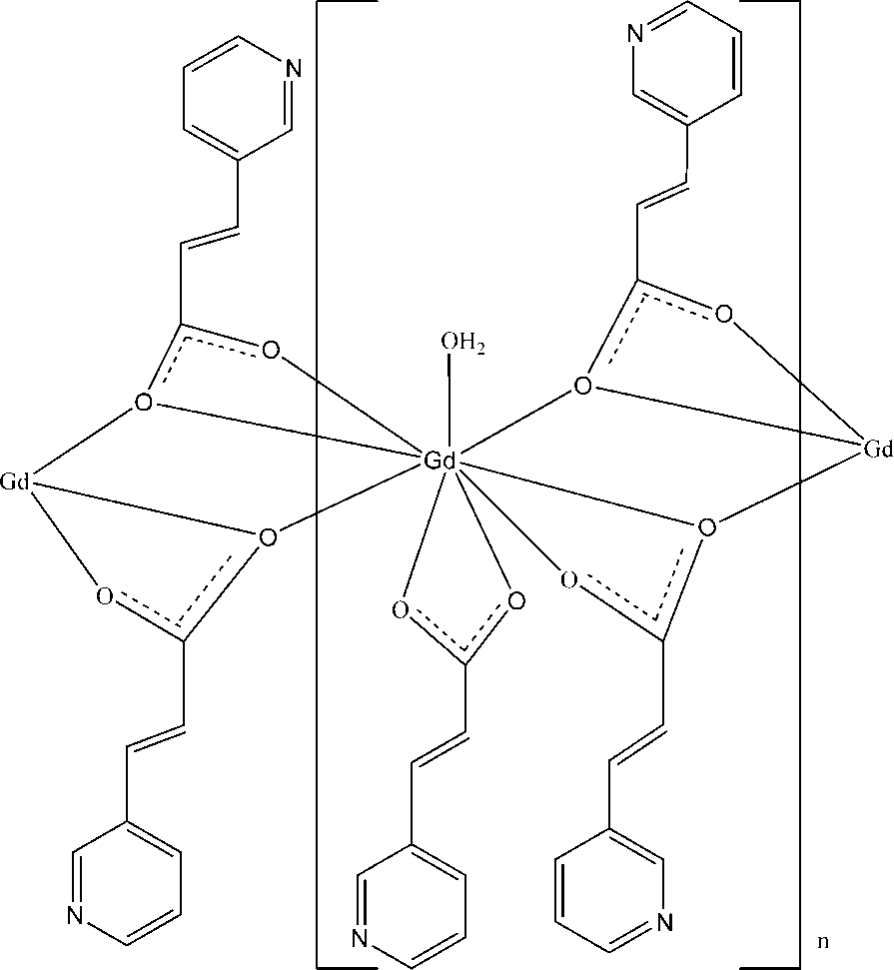


## Experimental

### 

#### Crystal data

[Gd(C_8_H_6_NO_2_)_3_(H_2_O)]
*M* *_r_* = 619.68
Triclinic, 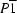

*a* = 6.2118 (7) Å
*b* = 12.7222 (14) Å
*c* = 15.6601 (18) Å
α = 111.741 (2)°
β = 90.309 (2)°
γ = 95.260 (2)°
*V* = 1143.7 (2) Å^3^
*Z* = 2
Mo *K*α radiation
μ = 2.95 mm^−1^
*T* = 294 (2) K
0.24 × 0.16 × 0.10 mm

#### Data collection

Bruker SMART 1000 diffractometer
Absorption correction: multi-scan (*SADABS*; Bruker, 2007 ▶) *T*_min_ = 0.538, *T*_max_ = 0.757
5840 measured reflections
3998 independent reflections
3745 reflections with *I* > 2σ(*I*)
*R*_int_ = 0.024

#### Refinement

*R*[*F* ^2^ > 2σ(*F* ^2^)] = 0.027
*wR*(*F* ^2^) = 0.070
*S* = 1.04
3998 reflections
324 parameters
3 restraints
H atoms treated by a mixture of independent and constrained refinement
Δρ_max_ = 1.46 e Å^−3^
Δρ_min_ = −2.22 e Å^−3^

### 

Data collection: *SMART* (Bruker, 2007 ▶); cell refinement: *SAINT* (Bruker, 2007 ▶); data reduction: *SAINT*; program(s) used to solve structure: *SHELXS97* (Sheldrick, 2008 ▶); program(s) used to refine structure: *SHELXL97* (Sheldrick, 2008 ▶); molecular graphics: *SHELXTL* (Bruker, 2007 ▶); software used to prepare material for publication: *SHELXTL*.

## Supplementary Material

Crystal structure: contains datablocks I, global. DOI: 10.1107/S1600536808012981/om2225sup1.cif

Structure factors: contains datablocks I. DOI: 10.1107/S1600536808012981/om2225Isup2.hkl

Additional supplementary materials:  crystallographic information; 3D view; checkCIF report

## Figures and Tables

**Table 1:** Hydrogen-bond geometry (Å, °)

*D*—H⋯*A*	*D*—H	H⋯*A*	*D*⋯*A*	*D*—H⋯*A*
O7—H7*A*⋯N1^i^	0.853 (10)	1.891 (18)	2.727 (4)	166 (5)
O7—H7*B*⋯O3^ii^	0.850 (10)	1.908 (13)	2.752 (3)	172 (4)

Symmetry codes: (i) 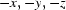; (ii) 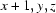.

**Fig. 1.**
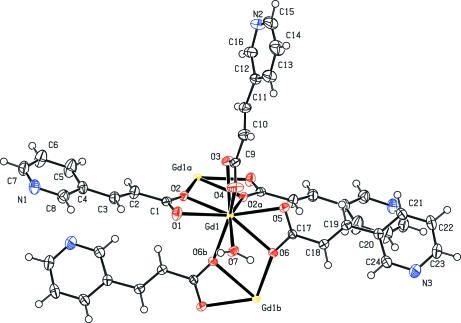
A portion of the structure showing the coordination environment of the GdIII ion, with displacement ellipsoids at the 50% probability level. H atoms are shown as small spheres of arbitrary radii. [Symmetry codes: (a) -x,1-y,-z; (b)1-x,1-y,-z.]

**Fig. 2.**
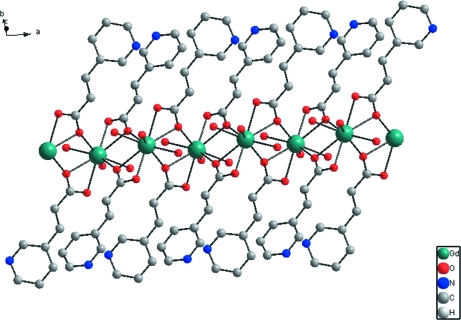
Part of a chain structure along the a axis. All H atoms are omitted.

**Fig. 3.**
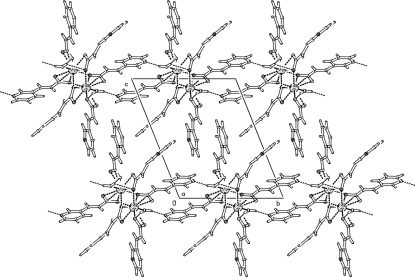
A packing diagram viewed along the a axis.

## supplementary crystallographic information

### Comment

The bifunctional ligand 3-pyridylacrylic acid (HTPA) is a potential multidentate ligand,and several types of complexes of HTPA have been studied (Ayyappan *et al.* ,2001; Gunning & Cahill, 2005; Zhang *et al.*, 2000). Until now, however, only a few crystallographic studies of 4*f*-block metal complexes of HTPA have been reported (Liu *et al.*, 2006; Liu *et al.*, 2004; Zhou *et al.*, 2004).

Here, we reported the synthesis and structure of the title complex, [Gd(TPA)_3_(H_2_O)]_n_ (Fig.1), whose structure consists of a repeating unit of formula [Gd(TPA)_3_(H_2_O)]. Each Gd^III^ centre is coordinated by eight carboxylate O atoms and one water molecule. The gadolinium(III) ions are joined into a coordination polymer chain along [100] (Fig.2), reinforced by O—H···O hydrogen bonds. Adjacent [100] chains are linked by O—H···N hydrogen bonds, forming sheets parallel to (001) (Fig.3).

### Experimental

A mixture of Gd(NO_3_)_3_^.^6H_2_O (0.25 mmol, 0.110 g), 3-pyridylacrylic acid (1.25 mmol,0.186 g), H_2_O (14 ml) was sealed in a 25 ml Teflon-lined stainless reactor and heated at 438 K for four days under autogenous pressure, then followed by slow cooling to room temperature, when a few colourless crystals were obtained. Analysis:found C 46.55, H 3.28, N 6.85%; C_24_H_20_GdN_3_O_7_ requires C 46.48, H 3.23, N 6.78%.

### Refinement

H atoms of the water molecules were located in a difference map and freely refined. H atoms bonded to C atoms were placed at calculated positions and treated using a riding-model approximation [C—H = 0.93Å and *U*_iso_(H)= 1.2*U*_eq_(C)].

### Crystal data

**Table:**

[Gd(C_8_H_6_NO_2_)_3_(H_2_O)]	*Z* = 2
*M**_r_* = 619.68	*F*_000_ = 610
Triclinic, *P*1	*D*_x_ = 1.799 Mg m^−^^3^
Hall symbol: -P 1	Mo *K*α radiation λ = 0.71073 Å
*a* = 6.2118 (7) Å	Cell parameters from 5157 reflections
*b* = 12.7222 (14) Å	θ = 2.7–25.9º
*c* = 15.6601 (18) Å	µ = 2.95 mm^−^^1^
α = 111.741 (2)º	*T* = 294 (2) K
β = 90.309 (2)º	Block, colourless
γ = 95.260 (2)º	0.24 × 0.16 × 0.10 mm
*V* = 1143.7 (2) Å^3^	

### Data collection

**Table:**

Bruker SMART 1000 diffractometer	3998 independent reflections
Radiation source: fine-focus sealed tube	3745 reflections with *I* > 2σ(*I*)
Monochromator: graphite	*R*_int_ = 0.024
*T* = 294(2) K	θ_max_ = 25.0º
φ and ω scans	θ_min_ = 1.7º
Absorption correction: multi-scan(SADABS; Bruker, 2007)	*h* = −3→7
*T*_min_ = 0.538, *T*_max_ = 0.757	*k* = −15→14
5840 measured reflections	*l* = −18→18

### Refinement

**Table:**

Refinement on *F*^2^	Secondary atom site location: difference Fourier map
Least-squares matrix: full	Hydrogen site location: inferred from neighbouring sites
*R*[*F*^2^ > 2σ(*F*^2^)] = 0.027	H atoms treated by a mixture of independent and constrained refinement
*wR*(*F*^2^) = 0.070	*w* = 1/[σ^2^(*F*_o_^2^) + (0.0466*P*)^2^] where *P* = (*F*_o_^2^ + 2*F*_c_^2^)/3
*S* = 1.04	(Δ/σ)_max_ = 0.001
3998 reflections	Δρ_max_ = 1.46 e Å^−^^3^
324 parameters	Δρ_min_ = −2.22 e Å^−^^3^
3 restraints	Extinction correction: none
Primary atom site location: structure-invariant direct methods	

### Special details

**Table:**

Geometry. All e.s.d.'s (except the e.s.d. in the dihedral angle between two l.s. planes) are estimated using the full covariance matrix. The cell e.s.d.'s are taken into account individually in the estimation of e.s.d.'s in distances, angles and torsion angles; correlations between e.s.d.'s in cell parameters are only used when they are defined by crystal symmetry. An approximate (isotropic) treatment of cell e.s.d.'s is used for estimating e.s.d.'s involving l.s. planes.
Refinement. Refinement of *F*^2^ against ALL reflections. The weighted *R*-factor *wR* and goodness of fit *S* are based on *F*^2^, conventional *R*-factors *R* are based on *F*, with *F* set to zero for negative *F*^2^. The threshold expression of *F*^2^ > σ(*F*^2^) is used only for calculating *R*-factors(gt) *etc*. and is not relevant to the choice of reflections for refinement. *R*-factors based on *F*^2^ are statistically about twice as large as those based on *F*, and *R*- factors based on ALL data will be even larger.

### Fractional atomic coordinates and isotropic or equivalent isotropic displacement parameters (Å^2^)

**Table:**

	*x*	*y*	*z*	*U*_iso_*/*U*_eq_	
Gd1	0.24371 (2)	0.472144 (12)	0.075317 (10)	0.01434 (8)	
O1	0.0775 (4)	0.2748 (2)	0.01956 (19)	0.0278 (6)	
O2	−0.0886 (4)	0.38753 (19)	−0.03041 (16)	0.0186 (5)	
O3	−0.0667 (4)	0.5066 (2)	0.17142 (16)	0.0234 (5)	
O4	0.1965 (4)	0.4402 (2)	0.22361 (19)	0.0347 (7)	
O5	0.4079 (4)	0.6447 (2)	0.19685 (17)	0.0253 (6)	
O6	0.5959 (4)	0.59101 (19)	0.07279 (15)	0.0192 (5)	
O7	0.5409 (4)	0.3842 (2)	0.10173 (19)	0.0235 (5)	
H7A	0.534 (8)	0.319 (2)	0.106 (4)	0.09 (2)*	
H7B	0.657 (4)	0.428 (3)	0.122 (3)	0.060 (17)*	
N1	−0.5448 (6)	−0.1952 (3)	−0.1445 (3)	0.0363 (9)	
N2	−0.6805 (6)	0.6840 (3)	0.5098 (3)	0.0473 (10)	
N3	1.2920 (6)	0.9676 (4)	0.3726 (3)	0.0581 (13)	
C1	−0.0758 (6)	0.2899 (3)	−0.0247 (2)	0.0202 (7)	
C2	−0.2482 (6)	0.1977 (3)	−0.0705 (3)	0.0270 (8)	
H2	−0.3645	0.2140	−0.0995	0.032*	
C3	−0.2458 (6)	0.0938 (3)	−0.0724 (3)	0.0281 (8)	
H3	−0.1240	0.0777	−0.0466	0.034*	
C4	−0.4190 (6)	0.0005 (3)	−0.1120 (3)	0.0263 (8)	
C5	−0.6180 (8)	0.0145 (4)	−0.1449 (4)	0.0452 (12)	
H5	−0.6448	0.0852	−0.1450	0.054*	
C6	−0.7752 (7)	−0.0755 (4)	−0.1773 (3)	0.0431 (11)	
H6	−0.9080	−0.0669	−0.2003	0.052*	
C7	−0.7328 (7)	−0.1786 (3)	−0.1750 (3)	0.0374 (10)	
H7	−0.8405	−0.2391	−0.1959	0.045*	
C8	−0.3917 (7)	−0.1075 (3)	−0.1140 (3)	0.0321 (9)	
H8	−0.2593	−0.1194	−0.0930	0.039*	
C9	0.0238 (6)	0.4859 (3)	0.2362 (3)	0.0224 (8)	
C10	−0.0715 (6)	0.5192 (3)	0.3273 (3)	0.0279 (8)	
H10	−0.0013	0.5053	0.3741	0.033*	
C11	−0.2553 (6)	0.5686 (3)	0.3448 (3)	0.0274 (8)	
H11	−0.3250	0.5776	0.2956	0.033*	
C12	−0.3588 (6)	0.6102 (3)	0.4332 (3)	0.0273 (8)	
C13	−0.2705 (7)	0.6138 (4)	0.5164 (3)	0.0357 (9)	
H13	−0.1330	0.5915	0.5193	0.043*	
C14	−0.3881 (8)	0.6504 (4)	0.5937 (3)	0.0455 (11)	
H14	−0.3319	0.6534	0.6498	0.055*	
C15	−0.5910 (8)	0.6829 (4)	0.5871 (3)	0.0507 (13)	
H15	−0.6707	0.7056	0.6399	0.061*	
C16	−0.5622 (7)	0.6482 (3)	0.4349 (3)	0.0351 (9)	
H16	−0.6203	0.6488	0.3802	0.042*	
C17	0.5793 (6)	0.6546 (3)	0.1569 (2)	0.0180 (7)	
C18	0.7606 (6)	0.7385 (3)	0.2060 (3)	0.0228 (8)	
H18	0.8965	0.7308	0.1811	0.027*	
C19	0.7388 (6)	0.8243 (3)	0.2838 (3)	0.0257 (8)	
H19	0.6023	0.8298	0.3083	0.031*	
C20	0.9125 (6)	0.9118 (3)	0.3352 (3)	0.0283 (8)	
C21	0.8659 (7)	1.0191 (3)	0.3920 (3)	0.0363 (10)	
H21	0.7233	1.0366	0.3989	0.044*	
C22	1.0313 (7)	1.0991 (4)	0.4378 (3)	0.0458 (12)	
H22	1.0029	1.1718	0.4760	0.055*	
C23	1.2396 (8)	1.0707 (5)	0.4266 (4)	0.0546 (14)	
H23	1.3508	1.1259	0.4581	0.066*	
C24	1.1281 (7)	0.8911 (4)	0.3285 (3)	0.0426 (11)	
H24	1.1605	0.8189	0.2908	0.051*	

### Atomic displacement parameters (Å^2^)

**Table:**

	*U*^11^	*U*^22^	*U*^33^	*U*^12^	*U*^13^	*U*^23^
Gd1	0.01095 (11)	0.01488 (11)	0.01579 (12)	0.00058 (7)	0.00181 (7)	0.00424 (8)
O1	0.0259 (14)	0.0208 (14)	0.0372 (16)	−0.0015 (11)	−0.0083 (12)	0.0124 (12)
O2	0.0159 (12)	0.0158 (12)	0.0231 (13)	−0.0012 (9)	0.0005 (10)	0.0065 (10)
O3	0.0171 (12)	0.0325 (14)	0.0211 (13)	0.0011 (10)	0.0028 (10)	0.0109 (11)
O4	0.0255 (14)	0.0538 (19)	0.0321 (15)	0.0163 (13)	0.0089 (12)	0.0217 (14)
O5	0.0185 (13)	0.0281 (14)	0.0229 (13)	−0.0029 (11)	0.0045 (10)	0.0031 (11)
O6	0.0182 (12)	0.0216 (13)	0.0139 (12)	0.0015 (10)	0.0024 (9)	0.0023 (10)
O7	0.0142 (12)	0.0223 (14)	0.0372 (15)	0.0032 (10)	0.0010 (11)	0.0146 (12)
N1	0.043 (2)	0.0181 (17)	0.047 (2)	−0.0042 (15)	0.0030 (18)	0.0124 (16)
N2	0.035 (2)	0.049 (2)	0.045 (2)	0.0078 (18)	0.0182 (18)	0.0026 (19)
N3	0.0234 (19)	0.050 (3)	0.067 (3)	−0.0048 (18)	−0.0035 (19)	−0.015 (2)
C1	0.0186 (18)	0.0181 (18)	0.0231 (19)	0.0018 (14)	0.0052 (15)	0.0069 (15)
C2	0.0251 (19)	0.021 (2)	0.034 (2)	−0.0027 (15)	−0.0062 (17)	0.0105 (17)
C3	0.0260 (19)	0.022 (2)	0.033 (2)	0.0007 (15)	−0.0018 (16)	0.0066 (16)
C4	0.031 (2)	0.0178 (18)	0.028 (2)	−0.0019 (15)	−0.0004 (16)	0.0072 (15)
C5	0.045 (3)	0.027 (2)	0.069 (3)	−0.0043 (19)	−0.016 (2)	0.026 (2)
C6	0.037 (2)	0.035 (2)	0.058 (3)	−0.0075 (19)	−0.016 (2)	0.021 (2)
C7	0.040 (2)	0.030 (2)	0.035 (2)	−0.0119 (18)	0.0007 (19)	0.0070 (18)
C8	0.031 (2)	0.024 (2)	0.042 (2)	0.0014 (17)	−0.0012 (18)	0.0132 (18)
C9	0.0168 (17)	0.0264 (19)	0.0230 (19)	−0.0014 (15)	0.0026 (14)	0.0088 (16)
C10	0.027 (2)	0.038 (2)	0.0214 (19)	0.0061 (17)	0.0027 (15)	0.0128 (17)
C11	0.0258 (19)	0.032 (2)	0.024 (2)	0.0024 (16)	0.0057 (16)	0.0098 (17)
C12	0.028 (2)	0.027 (2)	0.024 (2)	0.0011 (16)	0.0069 (16)	0.0054 (16)
C13	0.037 (2)	0.039 (2)	0.031 (2)	0.0079 (18)	0.0056 (18)	0.0119 (19)
C14	0.061 (3)	0.051 (3)	0.022 (2)	0.002 (2)	0.007 (2)	0.011 (2)
C15	0.054 (3)	0.047 (3)	0.036 (3)	−0.001 (2)	0.024 (2)	0.000 (2)
C16	0.028 (2)	0.040 (2)	0.032 (2)	0.0078 (18)	0.0064 (17)	0.0066 (19)
C17	0.0181 (17)	0.0150 (17)	0.0187 (18)	0.0030 (13)	−0.0004 (14)	0.0034 (14)
C18	0.0191 (18)	0.0217 (19)	0.025 (2)	0.0000 (14)	0.0040 (15)	0.0059 (16)
C19	0.0176 (17)	0.027 (2)	0.027 (2)	0.0020 (15)	0.0016 (15)	0.0039 (16)
C20	0.0223 (19)	0.024 (2)	0.029 (2)	−0.0014 (15)	0.0008 (16)	−0.0001 (16)
C21	0.028 (2)	0.027 (2)	0.039 (2)	0.0052 (17)	0.0007 (18)	−0.0059 (18)
C22	0.042 (3)	0.024 (2)	0.051 (3)	−0.0021 (19)	0.000 (2)	−0.008 (2)
C23	0.034 (3)	0.039 (3)	0.062 (4)	−0.012 (2)	−0.001 (2)	−0.011 (2)
C24	0.026 (2)	0.033 (2)	0.047 (3)	0.0035 (18)	−0.0005 (19)	−0.011 (2)

### Geometric parameters (Å, °)

**Table:**

Gd1—O7	2.354 (2)	C5—C6	1.371 (6)
Gd1—O6^i^	2.410 (2)	C5—H5	0.9300
Gd1—O2^ii^	2.418 (2)	C6—C7	1.373 (6)
Gd1—O3	2.424 (2)	C6—H6	0.9300
Gd1—O5	2.440 (2)	C7—H7	0.9300
Gd1—O1	2.454 (2)	C8—H8	0.9300
Gd1—O4	2.515 (3)	C9—C10	1.474 (5)
Gd1—O2	2.529 (2)	C10—C11	1.335 (5)
Gd1—O6	2.552 (2)	C10—H10	0.9300
O1—C1	1.245 (5)	C11—C12	1.462 (5)
O2—C1	1.287 (4)	C11—H11	0.9300
O2—Gd1^ii^	2.418 (2)	C12—C16	1.390 (6)
O3—C9	1.278 (4)	C12—C13	1.394 (6)
O4—C9	1.249 (4)	C13—C14	1.367 (6)
O5—C17	1.258 (4)	C13—H13	0.9300
O6—C17	1.276 (4)	C14—C15	1.377 (7)
O6—Gd1^i^	2.410 (2)	C14—H14	0.9300
O7—H7A	0.853 (10)	C15—H15	0.9300
O7—H7B	0.850 (10)	C16—H16	0.9300
N1—C7	1.323 (6)	C17—C18	1.470 (5)
N1—C8	1.336 (5)	C18—C19	1.319 (5)
N2—C15	1.334 (7)	C18—H18	0.9300
N2—C16	1.341 (5)	C19—C20	1.469 (5)
N3—C24	1.331 (6)	C19—H19	0.9300
N3—C23	1.342 (7)	C20—C21	1.384 (5)
C1—C2	1.475 (5)	C20—C24	1.385 (6)
C2—C3	1.313 (5)	C21—C22	1.366 (6)
C2—H2	0.9300	C21—H21	0.9300
C3—C4	1.471 (5)	C22—C23	1.370 (7)
C3—H3	0.9300	C22—H22	0.9300
C4—C8	1.388 (5)	C23—H23	0.9300
C4—C5	1.388 (6)	C24—H24	0.9300
			
O7—Gd1—O6^i^	77.79 (9)	O2—C1—C2	118.4 (3)
O7—Gd1—O2^ii^	151.90 (8)	O1—C1—Gd1	58.48 (19)
O6^i^—Gd1—O2^ii^	86.86 (8)	O2—C1—Gd1	62.02 (18)
O7—Gd1—O3	123.56 (9)	C2—C1—Gd1	176.2 (3)
O6^i^—Gd1—O3	151.93 (8)	C3—C2—C1	123.3 (4)
O2^ii^—Gd1—O3	79.63 (8)	C3—C2—H2	118.3
O7—Gd1—O5	86.21 (9)	C1—C2—H2	118.3
O6^i^—Gd1—O5	118.78 (8)	C2—C3—C4	125.7 (4)
O2^ii^—Gd1—O5	80.71 (8)	C2—C3—H3	117.2
O3—Gd1—O5	83.35 (8)	C4—C3—H3	117.2
O7—Gd1—O1	81.11 (9)	C8—C4—C5	116.4 (4)
O6^i^—Gd1—O1	82.79 (9)	C8—C4—C3	120.0 (4)
O2^ii^—Gd1—O1	120.49 (8)	C5—C4—C3	123.5 (3)
O3—Gd1—O1	83.09 (9)	C6—C5—C4	120.2 (4)
O5—Gd1—O1	152.02 (9)	C6—C5—H5	119.9
O7—Gd1—O4	71.15 (9)	C4—C5—H5	119.9
O6^i^—Gd1—O4	145.74 (8)	C5—C6—C7	118.8 (4)
O2^ii^—Gd1—O4	127.39 (8)	C5—C6—H6	120.6
O3—Gd1—O4	52.65 (8)	C7—C6—H6	120.6
O5—Gd1—O4	73.78 (9)	N1—C7—C6	122.7 (4)
O1—Gd1—O4	78.53 (10)	N1—C7—H7	118.7
O7—Gd1—O2	129.92 (8)	C6—C7—H7	118.7
O6^i^—Gd1—O2	79.03 (8)	N1—C8—C4	123.7 (4)
O2^ii^—Gd1—O2	68.19 (8)	N1—C8—H8	118.2
O3—Gd1—O2	73.13 (8)	C4—C8—H8	118.2
O5—Gd1—O2	143.61 (8)	O4—C9—O3	120.4 (3)
O1—Gd1—O2	52.30 (8)	O4—C9—C10	119.5 (3)
O4—Gd1—O2	110.31 (8)	O3—C9—C10	120.1 (3)
O7—Gd1—O6	70.08 (8)	O4—C9—Gd1	62.80 (19)
O6^i^—Gd1—O6	67.09 (9)	O3—C9—Gd1	58.70 (17)
O2^ii^—Gd1—O6	82.33 (8)	C10—C9—Gd1	167.6 (3)
O3—Gd1—O6	133.93 (8)	C11—C10—C9	122.2 (4)
O5—Gd1—O6	51.95 (8)	C11—C10—H10	118.9
O1—Gd1—O6	141.65 (8)	C9—C10—H10	118.9
O4—Gd1—O6	113.50 (8)	C10—C11—C12	126.9 (4)
O2—Gd1—O6	136.03 (7)	C10—C11—H11	116.5
O7—Gd1—C9	97.34 (10)	C12—C11—H11	116.5
O6^i^—Gd1—C9	165.27 (10)	C16—C12—C13	117.0 (4)
O2^ii^—Gd1—C9	102.81 (9)	C16—C12—C11	117.9 (4)
O3—Gd1—C9	26.77 (9)	C13—C12—C11	125.1 (4)
O5—Gd1—C9	74.26 (9)	C14—C13—C12	119.3 (4)
O1—Gd1—C9	82.72 (10)	C14—C13—H13	120.4
O4—Gd1—C9	26.20 (9)	C12—C13—H13	120.4
O2—Gd1—C9	94.12 (9)	C13—C14—C15	119.0 (5)
O6—Gd1—C9	124.69 (9)	C13—C14—H14	120.5
O7—Gd1—C1	105.52 (10)	C15—C14—H14	120.5
O6^i^—Gd1—C1	80.75 (9)	N2—C15—C14	124.1 (4)
O2^ii^—Gd1—C1	94.88 (9)	N2—C15—H15	117.9
O3—Gd1—C1	76.07 (9)	C14—C15—H15	117.9
O5—Gd1—C1	159.42 (9)	N2—C16—C12	124.6 (4)
O1—Gd1—C1	25.62 (10)	N2—C16—H16	117.7
O4—Gd1—C1	93.74 (10)	C12—C16—H16	117.7
O2—Gd1—C1	26.71 (9)	O5—C17—O6	119.6 (3)
O6—Gd1—C1	147.81 (9)	O5—C17—C18	120.8 (3)
O7—Gd1—C17	74.94 (9)	O6—C17—C18	119.7 (3)
O6^i^—Gd1—C17	93.45 (9)	O5—C17—Gd1	57.52 (18)
O2^ii^—Gd1—C17	82.75 (9)	O6—C17—Gd1	62.64 (17)
O3—Gd1—C17	108.93 (9)	C18—C17—Gd1	171.9 (3)
O5—Gd1—C17	25.77 (9)	C19—C18—C17	122.8 (3)
O1—Gd1—C17	156.00 (9)	C19—C18—H18	118.6
O4—Gd1—C17	92.19 (10)	C17—C18—H18	118.6
O2—Gd1—C17	150.21 (9)	C18—C19—C20	125.4 (3)
O6—Gd1—C17	26.36 (8)	C18—C19—H19	117.3
C1—O1—Gd1	95.9 (2)	C20—C19—H19	117.3
C1—O2—Gd1^ii^	156.8 (2)	C21—C20—C24	117.4 (4)
C1—O2—Gd1	91.3 (2)	C21—C20—C19	120.8 (3)
Gd1^ii^—O2—Gd1	111.81 (8)	C24—C20—C19	121.8 (3)
C9—O3—Gd1	94.5 (2)	C22—C21—C20	119.3 (4)
C9—O4—Gd1	91.0 (2)	C22—C21—H21	120.3
C17—O5—Gd1	96.7 (2)	C20—C21—H21	120.3
C17—O6—Gd1^i^	156.1 (2)	C21—C22—C23	119.0 (4)
C17—O6—Gd1	91.0 (2)	C21—C22—H22	120.5
Gd1^i^—O6—Gd1	112.91 (9)	C23—C22—H22	120.5
Gd1—O7—H7A	126 (3)	N3—C23—C22	123.6 (4)
Gd1—O7—H7B	116 (3)	N3—C23—H23	118.2
C7—N1—C8	118.2 (4)	C22—C23—H23	118.2
C15—N2—C16	116.0 (4)	N3—C24—C20	124.3 (4)
C24—N3—C23	116.3 (4)	N3—C24—H24	117.8
O1—C1—O2	120.4 (3)	C20—C24—H24	117.8
O1—C1—C2	121.2 (3)		
			
O7—Gd1—O1—C1	−162.3 (2)	O4—Gd1—C1—O2	130.10 (19)
O6^i^—Gd1—O1—C1	−83.6 (2)	O6—Gd1—C1—O2	−81.3 (2)
O2^ii^—Gd1—O1—C1	−1.6 (2)	C9—Gd1—C1—O2	104.69 (19)
O3—Gd1—O1—C1	72.0 (2)	O1—C1—C2—C3	−4.1 (6)
O5—Gd1—O1—C1	133.6 (2)	O2—C1—C2—C3	176.8 (4)
O4—Gd1—O1—C1	125.2 (2)	C1—C2—C3—C4	176.0 (4)
O2—Gd1—O1—C1	−1.9 (2)	C2—C3—C4—C8	176.6 (4)
O6—Gd1—O1—C1	−121.2 (2)	C2—C3—C4—C5	−6.4 (7)
C9—Gd1—O1—C1	99.0 (2)	C8—C4—C5—C6	−0.1 (7)
C17—Gd1—O1—C1	−165.9 (2)	C3—C4—C5—C6	−177.2 (4)
O7—Gd1—O2—C1	27.5 (2)	C4—C5—C6—C7	1.0 (8)
O6^i^—Gd1—O2—C1	91.20 (19)	C8—N1—C7—C6	0.6 (7)
O2^ii^—Gd1—O2—C1	−177.8 (2)	C5—C6—C7—N1	−1.3 (8)
O3—Gd1—O2—C1	−92.5 (2)	C7—N1—C8—C4	0.5 (6)
O5—Gd1—O2—C1	−144.5 (2)	C5—C4—C8—N1	−0.7 (6)
O1—Gd1—O2—C1	1.87 (19)	C3—C4—C8—N1	176.6 (4)
O4—Gd1—O2—C1	−54.5 (2)	Gd1—O4—C9—O3	12.0 (3)
O6—Gd1—O2—C1	130.66 (19)	Gd1—O4—C9—C10	−166.1 (3)
C9—Gd1—O2—C1	−75.6 (2)	Gd1—O3—C9—O4	−12.5 (4)
C17—Gd1—O2—C1	168.76 (19)	Gd1—O3—C9—C10	165.6 (3)
O7—Gd1—O2—Gd1^ii^	−154.70 (9)	O7—Gd1—C9—O4	−1.2 (2)
O6^i^—Gd1—O2—Gd1^ii^	−91.00 (10)	O6^i^—Gd1—C9—O4	68.4 (4)
O2^ii^—Gd1—O2—Gd1^ii^	0.0	O2^ii^—Gd1—C9—O4	−161.5 (2)
O3—Gd1—O2—Gd1^ii^	85.33 (10)	O3—Gd1—C9—O4	167.9 (4)
O5—Gd1—O2—Gd1^ii^	33.30 (17)	O5—Gd1—C9—O4	−85.2 (2)
O1—Gd1—O2—Gd1^ii^	179.67 (14)	O1—Gd1—C9—O4	78.8 (2)
O4—Gd1—O2—Gd1^ii^	123.32 (10)	O2—Gd1—C9—O4	129.9 (2)
O6—Gd1—O2—Gd1^ii^	−51.55 (14)	O6—Gd1—C9—O4	−72.0 (2)
C9—Gd1—O2—Gd1^ii^	102.16 (11)	C1—Gd1—C9—O4	104.1 (2)
C1—Gd1—O2—Gd1^ii^	177.8 (2)	C17—Gd1—C9—O4	−77.0 (2)
C17—Gd1—O2—Gd1^ii^	−13.4 (2)	O7—Gd1—C9—O3	−169.1 (2)
O7—Gd1—O3—C9	13.0 (2)	O6^i^—Gd1—C9—O3	−99.5 (4)
O6^i^—Gd1—O3—C9	147.8 (2)	O2^ii^—Gd1—C9—O3	30.6 (2)
O2^ii^—Gd1—O3—C9	−149.7 (2)	O5—Gd1—C9—O3	107.0 (2)
O5—Gd1—O3—C9	−68.0 (2)	O1—Gd1—C9—O3	−89.1 (2)
O1—Gd1—O3—C9	87.5 (2)	O4—Gd1—C9—O3	−167.9 (4)
O4—Gd1—O3—C9	6.7 (2)	O2—Gd1—C9—O3	−37.9 (2)
O2—Gd1—O3—C9	140.1 (2)	O6—Gd1—C9—O3	120.1 (2)
O6—Gd1—O3—C9	−81.1 (2)	C1—Gd1—C9—O3	−63.8 (2)
C1—Gd1—O3—C9	112.6 (2)	C17—Gd1—C9—O3	115.1 (2)
C17—Gd1—O3—C9	−71.1 (2)	O7—Gd1—C9—C10	102.6 (12)
O7—Gd1—O4—C9	178.7 (2)	O6^i^—Gd1—C9—C10	172.3 (11)
O6^i^—Gd1—O4—C9	−155.2 (2)	O2^ii^—Gd1—C9—C10	−57.6 (12)
O2^ii^—Gd1—O4—C9	22.9 (3)	O3—Gd1—C9—C10	−88.3 (13)
O3—Gd1—O4—C9	−6.8 (2)	O5—Gd1—C9—C10	18.7 (12)
O5—Gd1—O4—C9	87.2 (2)	O1—Gd1—C9—C10	−177.4 (12)
O1—Gd1—O4—C9	−96.9 (2)	O4—Gd1—C9—C10	103.9 (13)
O2—Gd1—O4—C9	−54.6 (2)	O2—Gd1—C9—C10	−126.2 (12)
O6—Gd1—O4—C9	121.5 (2)	O6—Gd1—C9—C10	31.8 (13)
C1—Gd1—O4—C9	−76.1 (2)	C1—Gd1—C9—C10	−152.0 (12)
C17—Gd1—O4—C9	105.5 (2)	C17—Gd1—C9—C10	26.8 (12)
O7—Gd1—O5—C17	62.5 (2)	O4—C9—C10—C11	179.8 (4)
O6^i^—Gd1—O5—C17	−11.4 (2)	O3—C9—C10—C11	1.7 (6)
O2^ii^—Gd1—O5—C17	−92.5 (2)	Gd1—C9—C10—C11	82.6 (13)
O3—Gd1—O5—C17	−173.1 (2)	C9—C10—C11—C12	−176.8 (4)
O1—Gd1—O5—C17	125.4 (2)	C10—C11—C12—C16	−173.3 (4)
O4—Gd1—O5—C17	134.0 (2)	C10—C11—C12—C13	6.1 (7)
O2—Gd1—O5—C17	−123.6 (2)	C16—C12—C13—C14	2.0 (6)
O6—Gd1—O5—C17	−5.1 (2)	C11—C12—C13—C14	−177.4 (4)
C9—Gd1—O5—C17	161.2 (2)	C12—C13—C14—C15	−0.1 (7)
C1—Gd1—O5—C17	−171.6 (2)	C16—N2—C15—C14	1.5 (7)
O7—Gd1—O6—C17	−96.3 (2)	C13—C14—C15—N2	−1.8 (8)
O6^i^—Gd1—O6—C17	179.0 (2)	C15—N2—C16—C12	0.6 (7)
O2^ii^—Gd1—O6—C17	89.1 (2)	C13—C12—C16—N2	−2.4 (6)
O3—Gd1—O6—C17	21.6 (2)	C11—C12—C16—N2	177.0 (4)
O5—Gd1—O6—C17	4.95 (19)	Gd1—O5—C17—O6	9.2 (4)
O1—Gd1—O6—C17	−139.9 (2)	Gd1—O5—C17—C18	−170.7 (3)
O4—Gd1—O6—C17	−38.4 (2)	Gd1^i^—O6—C17—O5	168.9 (4)
O2—Gd1—O6—C17	136.33 (19)	Gd1—O6—C17—O5	−8.7 (3)
C9—Gd1—O6—C17	−11.2 (2)	Gd1^i^—O6—C17—C18	−11.2 (8)
C1—Gd1—O6—C17	176.11 (18)	Gd1—O6—C17—C18	171.2 (3)
O7—Gd1—O6—Gd1^i^	84.78 (11)	Gd1^i^—O6—C17—Gd1	177.6 (6)
O6^i^—Gd1—O6—Gd1^i^	0.0	O7—Gd1—C17—O5	−113.6 (2)
O2^ii^—Gd1—O6—Gd1^i^	−89.82 (10)	O6^i^—Gd1—C17—O5	170.0 (2)
O3—Gd1—O6—Gd1^i^	−157.36 (9)	O2^ii^—Gd1—C17—O5	83.7 (2)
O5—Gd1—O6—Gd1^i^	−174.01 (14)	O3—Gd1—C17—O5	7.3 (2)
O1—Gd1—O6—Gd1^i^	41.09 (17)	O1—Gd1—C17—O5	−110.0 (3)
O4—Gd1—O6—Gd1^i^	142.62 (9)	O4—Gd1—C17—O5	−43.8 (2)
O2—Gd1—O6—Gd1^i^	−42.63 (14)	O2—Gd1—C17—O5	96.2 (3)
C9—Gd1—O6—Gd1^i^	169.87 (10)	O6—Gd1—C17—O5	171.0 (3)
C1—Gd1—O6—Gd1^i^	−2.8 (2)	C9—Gd1—C17—O5	−18.3 (2)
C17—Gd1—O6—Gd1^i^	−179.0 (2)	O7—Gd1—C17—O6	75.42 (19)
Gd1—O1—C1—O2	3.5 (4)	O6^i^—Gd1—C17—O6	−1.0 (2)
Gd1—O1—C1—C2	−175.5 (3)	O2^ii^—Gd1—C17—O6	−87.35 (19)
Gd1^ii^—O2—C1—O1	−178.2 (4)	O3—Gd1—C17—O6	−163.73 (18)
Gd1—O2—C1—O1	−3.4 (3)	O5—Gd1—C17—O6	−171.0 (3)
Gd1^ii^—O2—C1—C2	0.9 (8)	O1—Gd1—C17—O6	79.0 (3)
Gd1—O2—C1—C2	175.7 (3)	O4—Gd1—C17—O6	145.23 (19)
Gd1^ii^—O2—C1—Gd1	−174.8 (6)	O2—Gd1—C17—O6	−74.8 (3)
O7—Gd1—C1—O1	18.1 (2)	C9—Gd1—C17—O6	170.73 (19)
O6^i^—Gd1—C1—O1	92.6 (2)	O5—C17—C18—C19	−16.9 (6)
O2^ii^—Gd1—C1—O1	178.6 (2)	O6—C17—C18—C19	163.2 (4)
O3—Gd1—C1—O1	−103.3 (2)	C17—C18—C19—C20	−178.9 (4)
O5—Gd1—C1—O1	−104.9 (3)	C18—C19—C20—C21	153.6 (4)
O4—Gd1—C1—O1	−53.3 (2)	C18—C19—C20—C24	−26.0 (7)
O2—Gd1—C1—O1	176.6 (3)	C24—C20—C21—C22	0.5 (7)
O6—Gd1—C1—O1	95.3 (3)	C19—C20—C21—C22	−179.1 (4)
C9—Gd1—C1—O1	−78.7 (2)	C20—C21—C22—C23	−0.3 (8)
O7—Gd1—C1—O2	−158.44 (18)	C24—N3—C23—C22	0.1 (9)
O6^i^—Gd1—C1—O2	−83.95 (19)	C21—C22—C23—N3	−0.1 (10)
O2^ii^—Gd1—C1—O2	2.1 (2)	C23—N3—C24—C20	0.1 (9)
O3—Gd1—C1—O2	80.09 (19)	C21—C20—C24—N3	−0.4 (8)
O5—Gd1—C1—O2	78.6 (3)	C19—C20—C24—N3	179.2 (5)
O1—Gd1—C1—O2	−176.6 (3)		

Symmetry codes: (i) −*x*+1, −*y*+1, −*z*; (ii) −*x*, −*y*+1, −*z*.

### Hydrogen-bond geometry (Å, °)

**Table:**

*D*—H···*A*	*D*—H	H···*A*	*D*···*A*	*D*—H···*A*
O7—H7A···N1^iii^	0.853 (10)	1.891 (18)	2.727 (4)	166 (5)
O7—H7B···O3^iv^	0.850 (10)	1.908 (13)	2.752 (3)	172 (4)

Symmetry codes: (iii) −*x*, −*y*, −*z*; (iv) *x*+1, *y*, *z*.
